# Noninvasive reconstruction of cardiac electrical activity: update on current methods, applications and challenges

**DOI:** 10.1007/s12471-015-0690-9

**Published:** 2015-04-21

**Authors:** M.J.M. Cluitmans, R.L.M. Peeters, R.L. Westra, P.G.A. Volders

**Affiliations:** 1 Department of Cardiology, Cardiovascular Research Institute Maastricht, Maastricht University Medical Centre, PO Box 5800, 6202 AZ Maastricht, The Netherlands; 2 Department of Knowledge Engineering, Maastricht University, PO Box 616, 6200 MD Maastricht, The Netherlands

**Keywords:** Electrocardiography, Cardiac electrophysiology, Body surface potential mapping, Inverse problem of electrocardiography, Noninvasive electrocardiographic imaging

## Abstract

Electrical activity at the level of the heart muscle can be noninvasively reconstructed from body-surface electrocardiograms (ECGs) and patient-specific torso-heart geometry. This modality, coined electrocardiographic imaging, could fill the gap between the noninvasive (low-resolution) 12-lead ECG and invasive (high-resolution) electrophysiology studies. Much progress has been made to establish electrocardiographic imaging, and clinical studies appear with increasing frequency. However, many assumptions and model choices are involved in its execution, and only limited validation has been performed. In this article, we will discuss the technical details, clinical applications and current limitations of commonly used methods in electrocardiographic imaging. It is important for clinicians to realise the influence of certain assumptions and model choices for correct and careful interpretation of the results. This, in combination with more extensive validation, will allow for exploitation of the full potential of noninvasive electrocardiographic imaging as a powerful clinical tool to expedite diagnosis, guide therapy and improve risk stratification.

## Introduction

The 12-lead electrocardiogram (ECG) is a well-established, patient-friendly, quick, reproducible and cheap tool to determine normal cardiac activation and repolarisation to diagnose cardiac arrhythmias, altered activation, ischaemia, infarction, primary electrical abnormalities of the heart, structural disease, metabolic disorders, electrolyte imbalance and other conditions. It reflects the attenuated and dispersed result of propagated electrical activity and recovery in the heart on the body surface. However, it lacks the capacity to directly assess electrical activity at the level of the myocardium at high resolution. A modality that noninvasively images electrical activation and recovery at the heart surface could fill the gap between the noninvasive (low-resolution) 12-lead ECG and invasive (high-resolution) electrophysiology studies (EPs). Among other applications, this could facilitate the characterisation and imaging of electrical gradients under physiological and pathological conditions to localise the origins and circuits of ventricular tachycardias (VTs), to determine the substrate complexity underlying atrial fibrillation (AF) and to assess the size and location of an infarct scar, which are important for risk stratification.

In this regard, a current challenge is the *inverse problem of electrocardiography*, which is the main topic of this article. The inverse problem of electrocardiography indicates our current limitations to determine the electrical activity at the level of the heart muscle by means of body-surface ECGs and a patient-specific torso-heart geometry. The relation between electrical heart activity and its projection on the body surface depends on the torso-heart geometry and thoracic conductivities. The anatomical reference for a patient-specific torso-heart geometry can be provided by imaging modalities such as computed tomography (CT) or magnetic resonance imaging (MRI). The cardiac electrical activity can then be reconstructed in terms of local electrograms, depolarisation and repolarisation isochrones, activation sequences and other relevant electrophysiological signals.

Durrer et al. [1] were the first to thoroughly investigate the electrical activity of the intact human heart at the organ level. In their studies, they used explanted human hearts from patients who had died in the absence of heart disease. Years later, attempts to achieve this noninvasively in analytical and computer models [2–11], experimental dogs [12], torso-tank experiments with isolated canine hearts [13, 14] and humans [15, 16] were published.

During the past decades, much progress has been made in solving the inverse problem of electrocardiography [17–21] and applications in humans appear with increasing frequency [15, 16, 22]. Notably, Yoram Rudy’s group at Washington University in St. Louis, USA has published a diverse series of clinical studies in the past years (e.g. [23–29]). In previous investigations in the Netherlands, noninvasive reconstruction of electrical heart activity was studied as well [7, 30–34], and clinical collaborations will be pursued in the upcoming years [33, 35]. These methods carry different names, such as noninvasive electrocardiographic imaging (ECGI or ECG-imaging, coined by Yoram Rudy’s team) [15], myocardial activation imaging [22], electrocardiographic mapping [36], inverse solution mapping [16], noninvasive imaging of cardiac electrophysiology [37], three-dimensional cardiac activation imaging (3-DCAI) [38], or similar terminology. We will refer to this full range of techniques as ‘electrocardiographic imaging’.

**Fig. 1:**
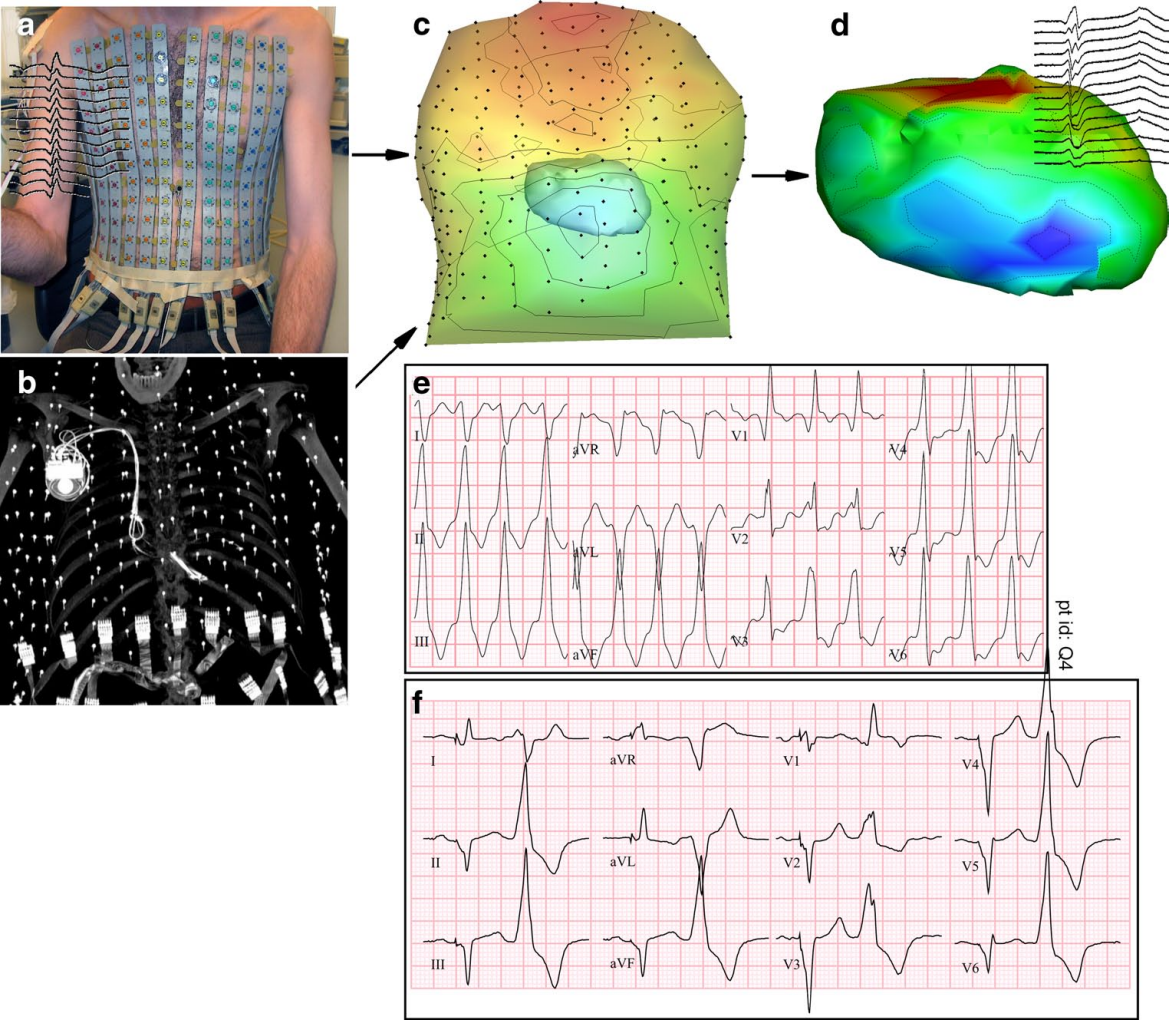
Inverse reconstruction of epicardial potentials in the patient from the text. Body-surface potentials are measured with 256 electrodes on the patient’s torso (**a**) and the geometrical and conductivity relationship between heart and body surface is, in this case, determined by computed tomography (**b**). The patient-specific inverse model (**c**) is then used to reconstruct epicardial potentials (**d**). Panel **e** shows the patient’s ventricular tachycardia, which shared morphology with his frequent ventricular extrasystoles (VES) beats (panel **f**: first beat paced, second beat VES). (Person in panel **a** is not the patient)

In the present paper, we will discuss the different techniques available to noninvasively reconstruct electrical heart activity based on body-surface ECG recordings and anatomical imaging. A patient case will illustrate the application of ECGI in our centre in Maastricht (Fig. 1). This patient, a 63-year-old man, was known with third-degree atrioventricular block, for which he received a pacemaker. After developing cardiomyopathy, his device was upgraded to a biventricular (BiV) pacemaker and implantable cardioverter-defibrillator. His heart failure worsened, and he developed a high count (20 %) of ventricular extrasystoles (VES). No genetic or ischaemic causes could be found and treatment with drugs proved insufficient. Ultrasound repeatedly confirmed a low left ventricular ejection fraction of 20 % and moderate mitral regurgitation. When readmitted with recurrent monomorphic sustained VT (VT morphology similar to his VES beats, see Fig. 1), an origin at the aortomitral continuity was suspected (based on the 12-lead ECG), but during the EP study, an epicardial origin close to the distal coronary sinus seemed more likely. The patient underwent ECGI before the EP study. His case will demonstrate how noninvasively reconstructed epicardial electrograms can help localise the origin of VT, and how reconstructed activation isochrones are superior to QRS duration and morphology on the regular 12-lead ECG for determining the electrical synchrony in cardiac resynchronisation therapy (CRT). In this paper, we will also point out current shortcomings and basic questions that need to be addressed before noninvasive ECGI can be fully accepted in the clinic. Understanding the methods and pitfalls of ECGI is essential for the correct interpretation of its results.

## Clinical applications

Applications of noninvasive ECGI are summarised in Table 1. One of these is the ability to detect the origin of VT [26, 39–42]. Although catheter ablation of sustained monomorphic VTs has been very successful in the past years, recurrence of VTs is still an issue, requiring repeated ablation procedures. There is not only a substantial recurrence rate in the large group of myocardial infarction-related VTs, but especially also in (inherited) cardiomyopathies [43]. Noninvasive ECGI may expedite EP studies and improve outcome, particularly in cases where the myocardium is very sensitive to catheter manipulation, or (such as the case presented in this paper) when only sporadic VES are present during the ablation procedure. These issues make intracardiac point-by-point mapping a strenuous task, whereas ECGI can be performed noninvasively for several hours and requires only one extrasystole to reconstruct its origin. Localising the origin of VT noninvasively can also reduce radiation burden and procedural complications and improve ablation success rate. For similar reasons, the management of atrial tachycardia may be facilitated by ECGI as well [44–46]. Furthermore, the suggested ability of ECGI to differentiate between an epicardial versus endocardial VT origin [40] would allow to select the appropriate ablation setup beforehand.

**Table 1:** Applications and method requirements for noninvasive reconstruction of electrical heart activity

Applications	Method requirements	References
VT and AT substrate	Sub-centimetre resolution for relatively large potentials; endocardial versus epicardial origin	[26, 39–42, 44–46]
AF substrate	Millimetre resolution for relatively small potentials	[25]
MI substrate	Detection of low-amplitude and fractionated electrograms	[27, 56]
CRT optimisation	Super-centimetre resolution for large potentials (electrical synchrony)	[36, 37, 47, 48]
Accessory pathways	Sub-centimetre resolution	[76, 77]
Repolarisation abnormalities	Centimetre resolution for relatively small potentials	[24, 28, 53–55]
Risk stratification (arrhythmogenesis)	Detection of conduction slowing and repolarisation abnormalities	[27, 56]

*VT*ventricular tachycardia, *AT* atrial tachycardia, *AF* atrial fibrillation, *MI* myocardial infarction, *CRT* cardiac resynchronisation therapy

ECGI may also help to reduce the currently large group of CRT non-responders, as it has been shown that noninvasive reconstruction of local activation timing predicts clinical CRT response better than QRS duration or the presence of left bundle branch block (LBBB) [36, 37, 47, 48]. Despite the low voltage amplitudes, AF has also been characterised by noninvasive reconstruction, assessing AF activation patterns and complexity [25], and ECGI significantly reduces invasive procedural time during AF ablation [49].

Another important public health problem is sudden cardiac death (SCD). Individual risk stratification for SCD is notably difficult [50]. ECGI may prove useful for the risk prediction of SCD by its ability to image and quantify conduction and repolarisation abnormalities, which are an important substrate for arrhythmias [24, 28, 51–55]. More specifically, the electro-anatomical characterisation of scar tissue after myocardial infarction, myocarditis or cardiomyopathies by assessing local low-amplitude and fractionated electrograms could contribute to risk stratification for VT and SCD [27, 56, 57].

Although all these examples illustrate the potential value of inverse electrocardiography, no specific application has been developed to such an extent yet that it has been included in a clinical guideline.

## Inverse reconstruction setup

**Fig. 2:**
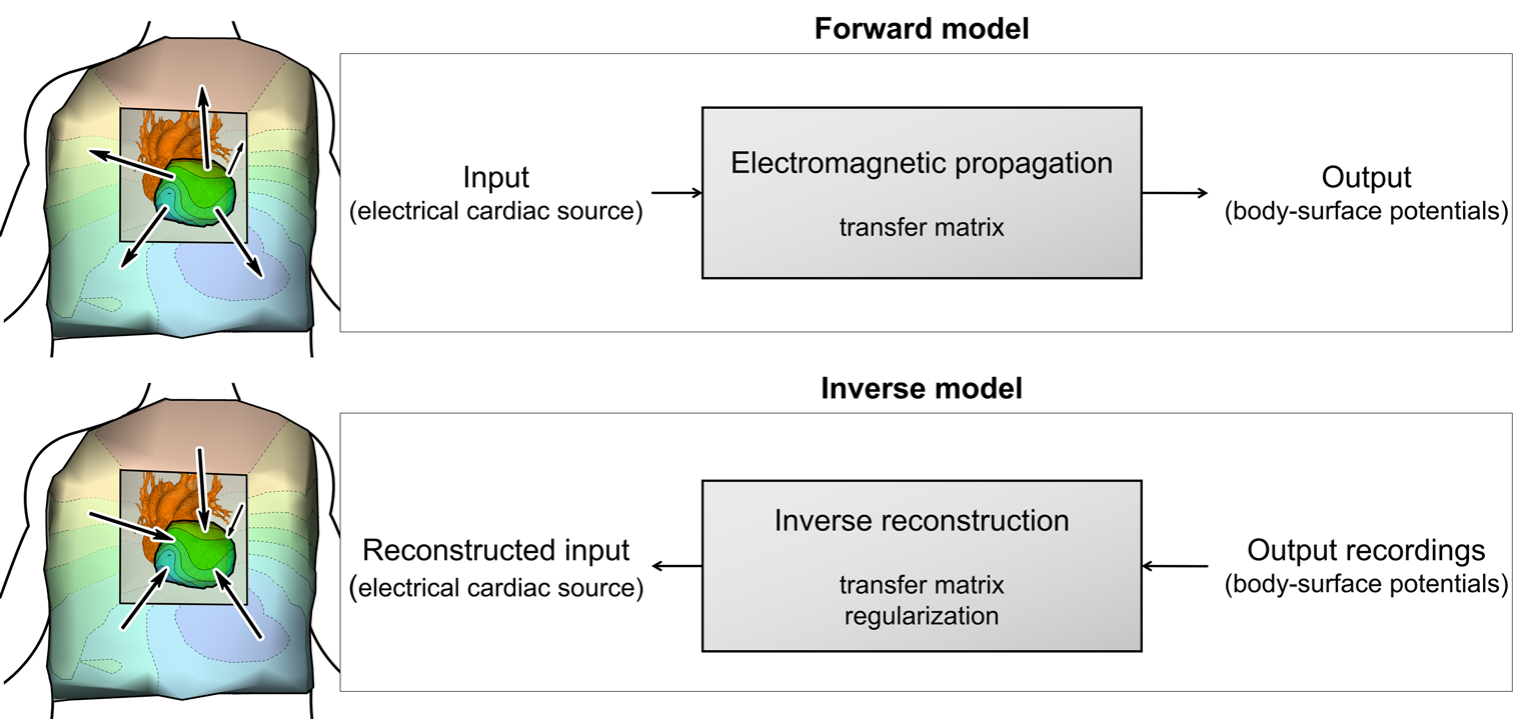
Schematic representation of forward/inverse models. A forward model describes the propagation of electromagnetic activity from the heart to the body surface; an inverse model reverses that relation, allowing for noninvasive reconstruction of electrical heart activity from measured body-surface potentials

The setup necessary for inverse reconstruction consists of specialised hardware and mathematical algorithms. So-called ‘forward models’ describe the propagation of electromagnetic activity from heart to body surface (Fig. 2). An inverse model is based on a forward model with known ‘output’ and unknown ‘source’; it essentially reverses the natural electromagnetic relationship between heart and body surface. Forward models consist of three parts: the cardiac source (representing the electrical activity of the heart), the model output (the body-surface potentials) and the electromagnetic source–output relation (capturing patient-specific propagation by an anatomical and conductivity reference). Reversing this electromagnetic relationship requires a fourth element, namely regularisation methods, to deal with the reconstruction’s sensitivity to noise. We will discuss these four elements in the next sections. Some technique characteristics are summarised in Table 2.

**Table 2:** Characteristics of commonly used methods for noninvasive reconstruction of electrical heart activity (details in text)

**General model assumptions**		
Volume conductor	(Piecewise) homogeneous: investigated in model studies but clinical implications unclear	
Breathing and cardiac motion	Assumed static: not investigated	
Torso volume	Heart as only electromagnetically active organ: not investigated	
**Specific model assumptions**		
	**Potential-based formulation**	**Wave-front formulation**
**Source representation**		
Source model	Epicardial potentials	Current dipole layers
Endocardial/epicardial	Only epicardial representation	Endo- and epicardial representation
**Output measurements (body-surface potentials)**		
Number and positioning of body-surface electrodes	Not established, 100 + electrodes used	Not established, 64 + electrodes used
**Regularisation and reconstruction**		
Sensitivity to ill-posedness (noise)	High	Intermediate
Local electrograms	Yes, direct	Yes, indirect
Epicardial information	Yes	Yes
Endocardial information	Maybe, under debate	Yes
Activation times	Yes, indirect	Yes, direct
Repolarisation times	Yes, indirect	Yes, direct
Fractionated potentials	Yes	No
Low amplitude potentials	Yes	No
Conduction slowing	Yes	Yes
**Validation**		
In vitro	Torso tank, extensive	None
Animal	None	Limited (only asynchronous, endocardial validation)
Human	Limited (asynchronous, open thorax or only endocardial)	Limited (asynchronous, only endocardial)
**Reconstruction quality**		
Detection of origin	Centimetre accuracy	Centimetre accuracy
Spatial resolution	Not investigated	Not investigated
Temporal resolution	Not investigated	Not investigated
**Clinical applications in literature**		
VT substrate	Yes	Yes
AT substrate	Yes	No
CRT optimisation	Yes	Yes
Accessory pathways	Yes	Yes
AF characterisation	Limited	No
Myocardial infarction	Limited	No
Repolarisation abnormalities	Limited	No

*VT* ventricular tachycardia, *AT* atrial tachycardia, *CRT* cardiac resynchronisation therapy, *AF* atrial fibrillation

### Body-surface potential measurements

To be able to reconstruct electrical activity of the heart, measurements of the projected potentials on the body surface should be taken at sufficient positions at anterior, posterior and lateral sides of the patient’s torso. These represent the ‘natural output’ of the electromagnetic (forward) propagation of cardiac electrical signals to the body surface. However, the minimum number of body-surface electrodes and their optimal positioning remains a subject of discussion, but is usually taken to be far more than the nine electrodes of the 12-lead ECG. Currently used setups include 64 [22], 120 [16] or 256 [26] electrodes. Simulation studies indicate that when a limited number of electrodes is used, their positioning is of significant influence on the quality of the inverse reconstructions [58]. Recently, the use of only the standard 12-lead ECG was shown to be worthwhile in some cases, but using this small number of body-surface electrodes has not been validated thoroughly [35]. We typically use 256 electrodes for body-surface potential measurements (Fig. 1a) with specialised hardware (BioSemi, Amsterdam, the Netherlands).

### Cardiac source representation

After measuring the body-surface potentials, a suitable model for the cardiac source must be chosen. Currently, two distinct representations for electrical heart activity are used most often: the *potential-based formulation* and the *wave-front formulation*. The potential-based formulation is built on the assumption that there is a direct and unique relation between the potentials at the epicardial surface and the potentials at the body surface, described by the conductivity properties of the torso as a passive electrical conductor [9, 15, 16, 36]. In this approach, local electrograms at the epicardium are reconstructed, from which additional information can be obtained, such as activation and repolarisation times, fractionated potentials, low-amplitude potentials, etc. Some approaches aim at reconstructing endocardial potentials at the same time, although there is still discussion about the feasibility of this approach [59, 60].

The wave-front formulation utilises different assumptions to model activation and repolarisation wave-fronts, by defining the wave-front (or more recently the complete endocardium and epicardium) as layer of current dipoles. [11, 22, 37, 38]. This approach is more restricted than the potential-based method, yielding only local activation and repolarisation times. On the other hand, the more sparse representation of the wave-front approach makes it less sensitive to noise. Furthermore, this method enables both endocardial and epicardial information to be obtained. In the approach applied by Van Oosterom’s group in the Netherlands, estimates for the cardiac source are used to solve the forward model to compute the corresponding body-surface potentials, which are then compared with the measured body-surface potentials [33]. Via an iterative approach, an optimal estimate of the activation sequence is computed to best match the measured body-surface potentials [61]. Recently, Van Oosterom [21] investigated the relation between the wave-front and potential-based methods and showed in a simulated case that also local electrograms can be computed from the wave-front formulation.

Other methods formulate the cardiac source fundamentally differently. For example, attempts were made to directly reconstruct areas of myocardial infarction [62]. Another method is to reconstruct transmembrane potentials, a ‘bidomain’ approach that connects computational models of intracellular and extracellular domains to body-surface potential measurements [63–65]. These approaches have not yet gained as much attention as the potential-based or wave-front formulation.

### Source-output relation: anatomical and conductivity reference

The next step in inverse reconstruction of electrical heart activity is determining the electromagnetic relation between source and output. This relation is captured by a patient-specific transfer matrix, based on conductivity and geometry of the patient’s torso. This is essential for creating accurate, patient-specific reconstructions that can be linked to anatomical location and abnormalities. A CT or MRI scan is used to obtain the anatomical reference (Fig. 1b), from which a geometry is created that consists of the heart surface, the location of the body-surface electrodes and the torso-volume conductor properties (Fig. 1c). The heart surface can either be defined purely by the epicardium (in the potential-based approach) or by both endocardium and epicardium (in the wave-front approach).

It is assumed that the torso volume can be divided into subregions with constant conductivity. In its simplest form, the human torso can be represented as completely homogeneous and containing only the heart. More realistic representations use a piecewise homogeneous torso, including other tissues such as the lungs, fatty tissue, bones and skeletal muscles, each with their specific conductivity. Some simulation studies suggest that the inclusion of torso inhomogeneities is necessary [21, 66], whereas others have revealed that the reconstructed epicardial potentials were only slightly less accurate when omitting inhomogeneities [67]. In vivo studies are needed to assess the clinical relevance including torso inhomogeneities in humans and whether it is relevant to create a more complicated geometry.

**Fig. 3:**
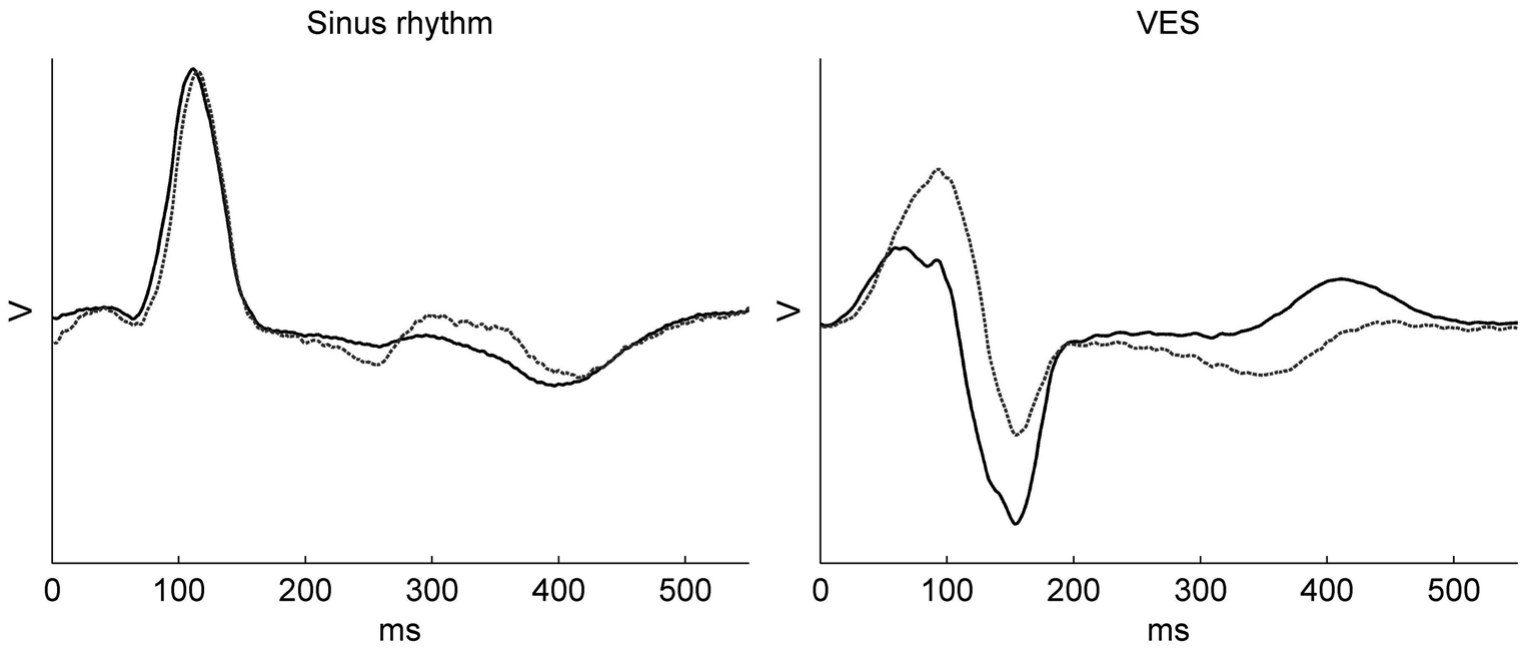
Reconstructed epicardial electrograms at one location of the left ventricle (indicated with an *asterisk* in Fig. 5) of a 63-year-old patient with native left bundle branch block (LBBB) during sinus rhythm and frequent ventricular extrasystoles (VES). In the reconstruction process, the same body-surface potentials (either sinus rhythm with LBBB or VES), but a different torso-heart geometry, were used: one with a diastolic cardiac geometry (resulting in the electrograms with a *solid line*), and one with a systolic cardiac geometry (*dashed line*). Using either a systolic or diastolic cardiac geometry results in significantly different electrograms on the same epicardial location, notably in the repolarisation phase. This indicates that cardiac contractile movement should be taken into account when reconstructing epicardial potentials. Voltage scales identical for both graphs

Another neglected element in the computation of the transfer matrix is the false assumption that both torso and heart are non-moving. The static torso-heart geometry is usually based on a CT scan taken during cardiac diastole and breath-hold, to capture both heart and torso in a quiescent state. However, during the recording of body-surface potentials that are used for inverse reconstruction, both torso and heart are changing in geometry (and conductivity). Especially, the reconstruction of repolarisation would suffer from the assumption of a static (diastolic) geometry, as we demonstrate for the patient in Fig. 3 where we used both a diastolic and a systolic geometry for reconstructing the same body-surface potentials on the epicardium. Clearly, the influence of using a static geometry should be investigated further in clinical studies.

Furthermore, the need for a CT or MRI scan hinders quick application of ECGI, complicates logistics in daily clinical practice and induces radiation burden to the patient when CT is used.

### Reconstruction and regularisation methods

After (1) measuring the body-surface potentials, (2) choosing a suitable cardiac source representation and (3) defining the electromagnetic relation between those two elements with a patient-specific transfer matrix, noninvasive reconstructions of cardiac electrical activity can be performed with an inverse model. However, the inverse problem is inherently *ill-posed*. This means that the solution of the problem is extremely sensitive to small perturbations in the measured body-surface potentials, such as noise. Ill-posedness is the mathematical consequence of the attenuating and dispersing effect of the electromagnetic propagation from heart to body surface [68]. Most implementations of the inverse problem, therefore, suffer from numerical instability. So-called ‘regularisation methods’ are needed to deal with this uncertainty and sensitivity in inverse models.

**Fig. 4:**
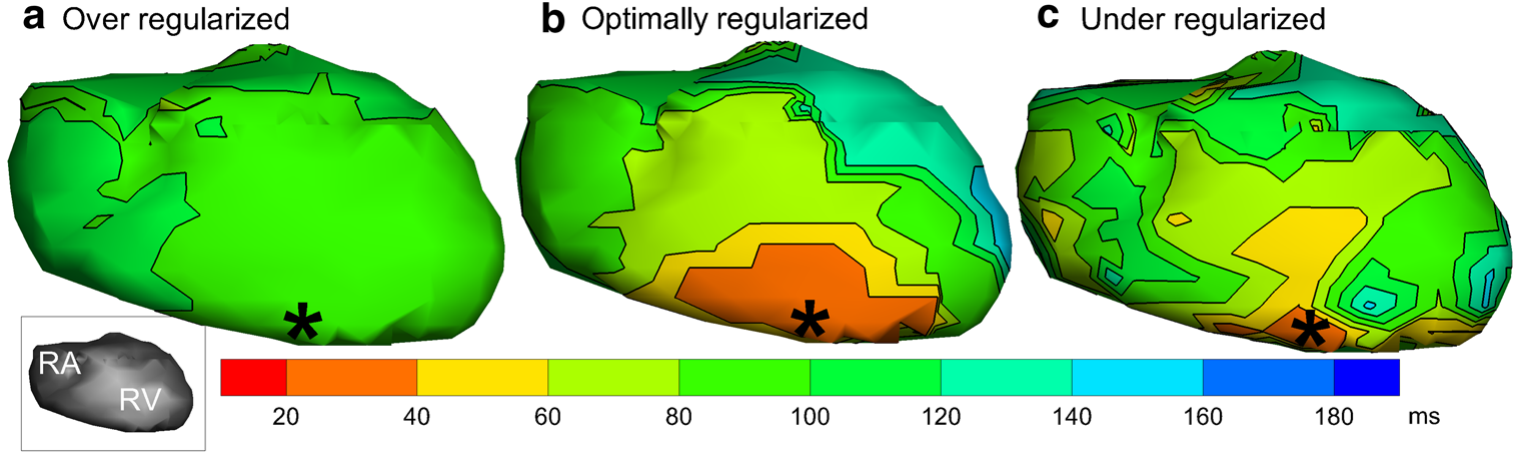
Noninvasively reconstructed isochrones of a paced beat, on the right ventricle (*RV*) of the same patient as in Fig. 3, for different values of the regularisation parameter. Panel **a** shows an over-regularised setting, applying the constraints too heavily, resulting in a reconstruction that does not contain any relevant information. An optimally regularised solution provides the most adequate reconstruction (panel **b**), showing early activation (*orange colour*) on the location of pacing (indicated with an *asterisk*). An under-regularised setting results in a reconstruction that is dominated by the influence of noise (panel **c**)

Regularisation incorporates additional knowledge in the inverse problem by applying constraints to the solutions, which will yield more realistic results. These additional constraints are based on physical or mathematical properties that apply to the forward/inverse models but that are not yet incorporated in the transfer matrix. When regularisation is applied, the weight of the constraints (the regularisation parameter) has to be determined to find a balance between solutions purely based on the body-surface potentials (possibly severely distorted by ill-posedness) and solutions that are constrained too strictly (possibly too much bias). Methods exist that aim at finding an optimal balance [69]. The important influence of the regularisation parameter is shown in Fig. 4 for the same patient as described previously.

The most commonly used regularisation method is Tikhonov regularisation, which is based on the idea that epicardial potentials should be reasonably small (zeroth order Tikhonov) or smoothly changing over the heart surface (first and second order Tikhonov) [70]. Other regularisation methods include truncated Singular Value Decomposition (tSVD) [71], Greensite SVD [72] and the Generalised Minimal Residual (GMRes) method [73]. Each method has its specific advantages and disadvantages, but all have difficulties with the inherent disadvantageous properties of the ill-posed inverse problem. Moreover, reconstruction quality can heavily depend on parameter settings. Recently, a first attempt was made to not only use physical and mathematical properties to regularise the solution, but to incorporate electrophysiological information as well to improve the quality of the reconstruction [74]. For an in-depth review of regularisation methods, we refer to Milanic et al. [75].

It is important for clinicians to realise that constraints are introduced purposefully to reach realistic solutions, but that this bias might create artefacts or prevent the reconstruction of electrical activity that is not accounted for by the constraints that were added.

## Reconstruction quality and clinical validation

**Fig. 5:**
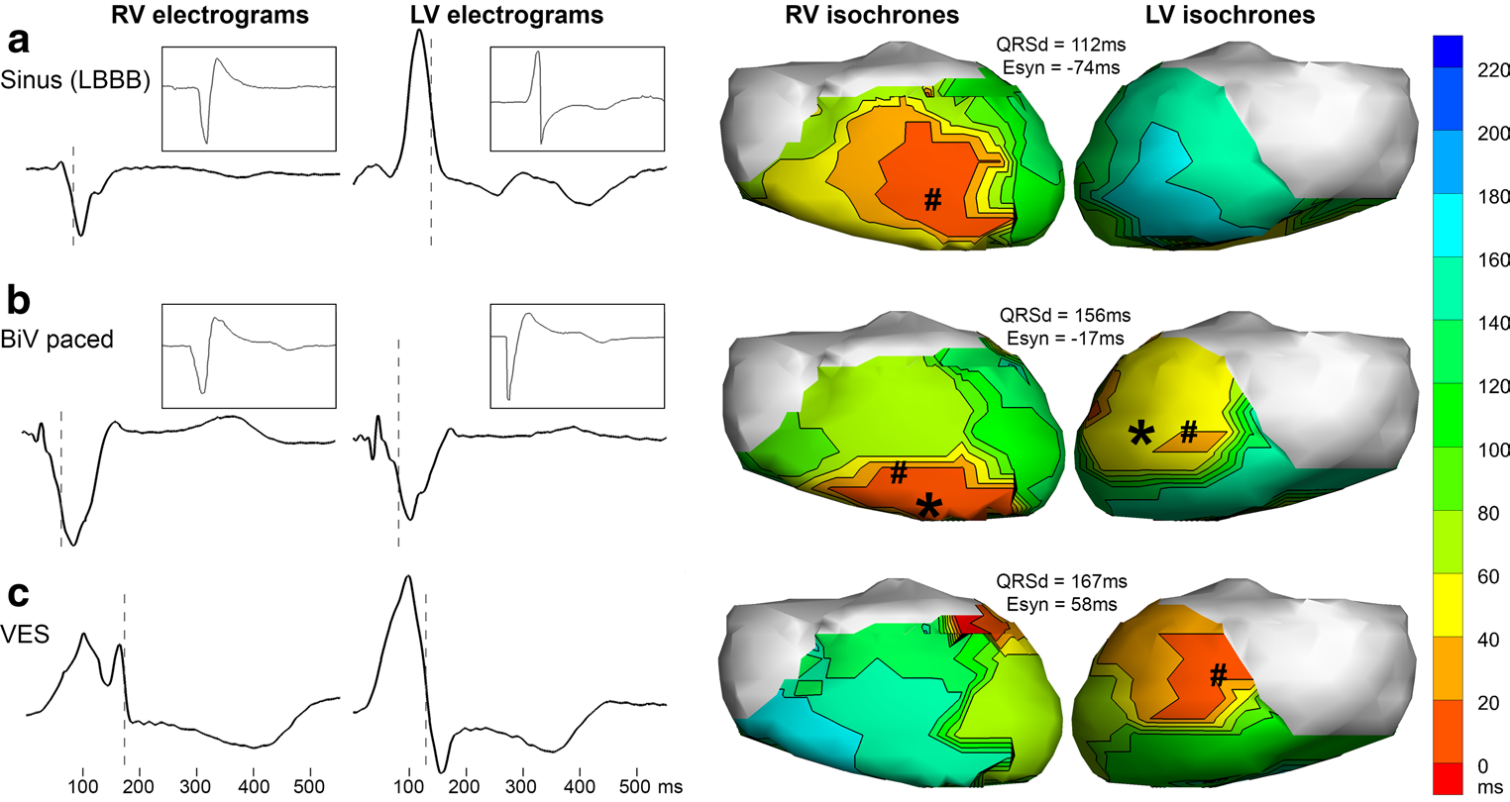
Reconstructed epicardial electrograms (*first two columns*, voltage scales identical over all graphs) and reconstructed activation isochrones (*last two columns*) for three different beats (row **a**: sinus beat with left bundle branch block pattern; **b**: biventricularly paced beat; **c**: extrasystolic beat). The reconstructed electrograms correspond to the epicardial location closest to the right and left pacing lead tips from the patient’s pacemaker. The insets show recorded electrograms from those leads at the same location for comparison, i.e. an endocardial recording for the right ventricle, and an epicardial recording for the left ventricle. The *dashed line* indicates the activation time (maximum − dV/dt). The ventricular activation isochrones, depicted in the *last two columns*, reflect the time of maximum − dV/dt per epicardial location. Locations of reconstructed earliest activation are indicated with symbol *#*. QRS duration (*QRSd*) is based on the 12-lead electrocardiography. Electrical synchrony (*Esyn*) is the difference between the mean activation times at the right ventricle (*RV*) and the mean activation times at the left ventricle (*LV*); a value close to zero usually results in more efficient contraction. For the paced beat (panel **b**), the pacing locations are indicated with symbol *

In the selected case from our patient studies, we obtained body-surface potentials with 256 electrodes and chose a potential-based representation of the cardiac source. The most important reason for doing so is to be able to reconstruct local electrograms and not only activation/repolarisation timing. Moreover, it has been validated more thoroughly in clinical settings. The patient-specific geometry, based on a CT scan during diastole, was chosen to be homogeneous. As a regularisation method, we applied Tikhonov zeroth order regularisation. Reconstructed electrograms are shown in Fig. 5, for three different beats in this patient. The insets in panel a and b show pseudo-unipolar electrograms recorded at the pacemaker lead tips in the right and left ventricle that correspond to the location of the shown reconstructed electrograms. Correlation coefficients between measured and reconstructed electrograms range from 0.79 to 0.85. Although the correlation coefficients are reasonably high, some important features are clearly not reconstructed correctly. This might be due to a combined effect of the pseudo-unipolar character of the pacemaker recordings, cardiac changes in the months between recording of body-surface potentials and pacemaker electrograms, the location mismatch in case of the right ventricle (endocardial recording versus epicardial reconstruction) and shortcomings in the inverse algorithm.

Activation isochrones can be created by determining the activation time at each epicardial location, as shown in the last two columns of Fig. 5. Activation of a sinus beat (panel a) starts at the right ventricle (red) and spreads to the left ventricle (blue), as was expected based on the LBBB morphology on the 12-lead ECG. Panel b shows activation isochrones for a paced beat (BiV paced). On the right ventricle, the reconstructed location with earliest activation (indicated with symbol #) is 23 mm from the known pacing location (indicated with symbol *) that was determined from the anatomical reference. For the left ventricle, the reconstructed and known locations of first activation are 28 mm apart. In agreement with pacemaker settings, the right ventricle was paced and activated before the left ventricle. The activation isochrones of a VES beat (panel c) suggest an origin of extrasystolic activity on the superior part of the left ventricle, although spatial resolution was too low to give a very precise location. From the EP study, an epicardial location at the mid-coronary sinus was suspected to be the origin, consistent with these reconstructions.

Figure 5 also demonstrates the advantage of inverse electrocardiography in CRT optimisation, by showing that electrical synchrony, i.e. measuring the time difference of activation of the complete left versus the complete right ventricle based on inversely reconstructed electrograms, gives better insight in ventricular (dys)synchrony than the often used 12-lead QRS duration.

Although reconstructed electrograms and activation times are consistent with expectations based on 12-lead ECGs, pacemaker recordings and the EP study, the level of accuracy and detail is suboptimal, with correlation coefficients being approximately 0.80 and a reconstructed pacing-origin mismatch of 23–28 mm. Other research groups have performed more extensive validation studies, claiming 10 mm accuracy and cross correlations of 0.70. [52] Other studies have found a significant decrease of accuracy in diseased hearts, with localisation accuracy going from 13 ± 9 mm (mean ± standard deviation) in healthy hearts up to 28 ± 27 mm in infarcted hearts and even 43 ± 11 mm near scarred tissue. [16] However, no thorough in vivo validation has been performed with potential recordings simultaneously at extensive locations at the heart and body surfaces in intact organisms.

## Limitations, added value and future potential

The potential applications of noninvasive reconstruction of electrical heart activity are promising. Localisation of extrasystolic activity in a noninvasive, per-beat and precise manner combined with an anatomical reference can expedite diagnosis, guide therapy and reduce procedural radiation burden. Electrical (dys)synchrony assessment allows patient-specific CRT and might help in improving therapy for current non-responders. Future applications could also include noninvasive risk stratification for conduction abnormalities (conduction slowing, electrical chaos in AF, low-voltage amplitudes), direct relation of structural abnormalities to electrical abnormalities (fibrosis, fibrofatty replacement) and easier visual interpretation of abnormalities that are already diagnosed on the powerful 12-lead ECG. Commercial, easy-to-use setups such as those developed by CardioInsight (Cleveland, OH, USA) will be essential to fully exploit the potential of these applications in daily clinical routine.

Whereas noninvasive reconstruction of electrical heart activity currently has a clear research potential, its added value in clinical practice remains to be established. It is still unclear when, from a patient and socioeconomic perspective, it is worth the extra effort of extensive mapping of body-surface potentials, performing a CT/MRI scan for anatomical reference, and carefully avoiding the regularisation and reconstruction pitfalls. More importantly, cardiologists should be aware of the existence and influence of those pitfalls and the assumptions underlying the reconstruction algorithms. Debate remains on topics such as the influence of inhomogeneities in the torso (notably the lungs) on the reconstructions, the optimal regularisation methods and their parameters, the maximum achievable resolution of reconstruction, the number and positioning of body-surface electrodes and the assumption of a static geometry. We feel that more extensive validation should be performed, besides additional clinical studies investigating inverse ECGI for various purposes. Extensive validation by in vivo studies, and careful interpretation of clinical results with knowledge about the underlying methods and assumptions, will allow full exploitation of the potential of noninvasive ECGI, improving diagnosis, therapy and risk stratification.
